# First principles study of optoelectronic and photocatalytic performance of novel transition metal dipnictide XP_2_ (X = Ti, Zr, Hf) monolayers

**DOI:** 10.1039/d2ra01851a

**Published:** 2022-04-11

**Authors:** Sheraz Ahmad, Ismail Shahid, Nasir Shehzad, W. Khan, H. U. Din, M. Idrees, B. Amin, A. Laref

**Affiliations:** School of Materials Science and Engineering, Computational Centre for Molecular Science, Institute of New Energy Material Chemistry, Nankai University Tianjin 300350 P. R. China; School of Physics, Nankai University Tianjin 300071 P. R. China; Department of Physics, Bacha Khan University Charsadda KP Pakistan haleem.uddin@yahoo.com; Department of Physics, Abbottabad University of Science & Technology Havelian Abbottabad KP Pakistan; Department of Physics and Astronomy, College of Science, King Saud University Riyadh 11451 Saudi Arabia

## Abstract

Low cost and highly efficient two dimensional materials as photocatalysts are gaining much attention to utilize solar energy for water splitting and produce hydrogen fuel as an alternative to deal with the energy crisis and reduce environmental hazards. First principles calculations are performed to investigate the electronic, optical and photocatalytic properties of novel two dimensional transition metal dipnictide XP_2_ (X = Ti, Zr, Hf) monolayers. The studied single layer XP_2_ is found to be dynamically and thermally stable. TiP_2_, ZrP_2_ and HfP_2_ systems exhibit semiconducting nature with moderate indirect band gap values of 1.72 eV, 1.43 eV and 2.02 eV, respectively. The solar light absorption is found to be in energy range of 1.65–3.3 eV. All three XP_2_ systems (at pH = 7) and the HfP_2_ monolayer (at pH = 0) that straddle the redox potentials, are promising candidates for the water splitting reaction. These findings enrich the two dimensional family and provide a platform to design novel devices for emerging optoelectronic and photovoltaic applications.

## Abstract 2

Low cost and highly efficient two dimensional materials as photocatalysts are gaining much attention to utilize solar energy for water splitting and produce hydrogen fuel as an alternative to deal with the energy crisis and reduce environmental hazards.

## Graphical abstract

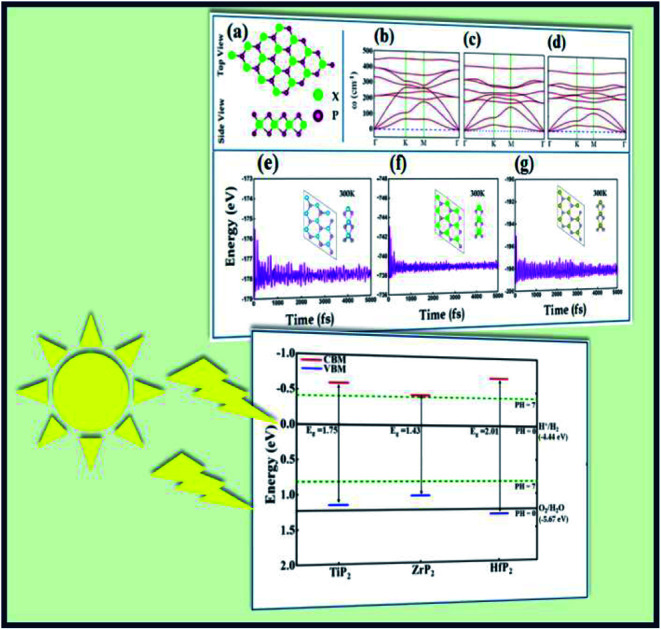

## Introduction

The reduction in fossil fuels and challenges to energy sources have forced the scientific society to search for environment friendly and efficient green energy fuels for sustainable development of a clean society.^1^ Hydrogen is considered as an ideal energy carrier source due to its abundance on earth, low pollution emission^2^ and highest energy per mass ratio. To produce hydrogen from water, photocatalytic water splitting is the most favorable method. For a few decades, researchers have been devoted to producing some novel metal-based inexpensive environment friendly photocatalytic materials.^3^ Two-dimensional materials like hexagonal boron nitride (h-BN) and transition metal dichalcogenides (TMDCs) have invoked considerable interest after the successful development of graphene in 2004.^4^ Single layer transition metal dichalcogenides have tremendous applications like optoelectronics, spintronics, valleytronics, photovoltaic devices, gas sensing, and catalysis due to their suitable band gap, good mechanical properties and absorption coefficients.^5–8^ The developed nano- and mesostructures, phosphides and phosphates have shown good flexibility and electrical conductivity^9^ in comparison with oxides and polymers, and are ideal for storing electrochemical energy based on faradaic redox reactions.^10^

Ranking 10^th^ in the abundance in the earth crust, phosphorus is considered very effective for hydrogen evolution^11^ and reports have shown that P played main role in photo catalysts.^12^ Due to low band gap, good stability and electrical conductivity, transition metal phosphides (TMPs) are considered as good semiconductor materials.^13^ Many compounds of phosphorus including phosphides and metal phosphates have been studied for super capacitors,^14^ lithium ion batteries^15^ and catalysts.^16^ Phosphorus make variety of phosphides when react with various elements in periodic table.^17^ For making transition metal phosphides, large atomic radius (0.109) of phosphorus makes it favorable in designing various crystal structures.^9^ Doping of some noble metals Pt, Pd, Au make photocatalytic material very efficient for photocatalytic hydrogen production^18,19^ but their applications are limited due to high cost.^20^ Therefore, search for more effective and cheap catalysts are very essential and highly demanding.^21^

Generally, two conditions must be fulfilled for photocatalytic material: (1) its conduction band edge should be more negative than H_2_ energy level, (2) the valence band edge must be lower than the energy level of oxygen.^22^ Despite of efficient performance of hydrogen evolution reaction (HER) for using catalyst in water splitting, controlling the basic structural composition is still a challenge.^23,24^ C. Y. Son and coworkers have synthetically developed FeP and FeP_2_ nanowires and demonstrated high electro-catalytic performance for P-rich FeP_2_ nanowires in acidic and basic solution. FeP_2_ revealed remarkable performance for water splitting. Transition metal phosphides have been discovered recently to decompose methyl orange efficiently and to produce hydrogen by photocatalytic activity.^25^ These findings have opened new windows for searching of novel photocatalysts and screening of new materials.^26,27^ Recent reports show MoP_2_ is used to produce hydrogen as semi-metallic photocatalyst. MoP_2_ nanosheets possess excellent electronic properties with high active site density exposure.^28^ Many early transition metal dipnictides (TMDPs) have been explored in orthorhombic and OsGe_2_ or MoP_2_ type-phase.^28–36^ However, TMDPs with hexagonal graphene-like structure remains unexplored.

In this paper, we theoretically studied structural, electronic, optical, and photocatalytic properties of novel 2D transition metal dipnictides XP_2_ (X = Ti, Zr, Hf). Our results show that XP_2_ (X = Ti, Zr, Hf) with stable graphene-like hexagonal geometry are semiconductors with moderate band gaps which show good optical activity in visible and near ultraviolet region. Furthermore, the band edge positions straddle the water redox potential, predicting XP_2_ monolayers as suitable candidates for photocatalytic water splitting.

## Computational details

The projector augmented plane wave (PAW)^37,38^ scheme was employed using density functional theory (DFT) implemented in Vienna *ab initio* simulation package (VASP).^39,40^ The electronic band structure is calculated using Perdew–Burke–Ernzerhof (PBE)^41^ functional. The semi-empirical van der Waals (vdW) corrections are considered as proposed by Grimme.^42,43^ We relaxed XP_2_ monolayers and converged energy to 10^−5^ eV and residual forces to 10^−4^ eV Å^−1^. A Γ-centered Monkhorst–Pack *k*-meshes of 6 × 6 × 1 were used for structural relaxation of monolayers and for optimized structures upgraded to 12 × 12 × 1 with 500 eV was used as plane–wave cutoff energy. A vacuum layer of 25 Å in direction of out-of-plane was used to preclude interaction between layers. The converged PBE wave functions were used as starting point of Heyd–Scuseria–Ernzerhof (HSE06).^44^ VASP associated phonopy code was used for phonon spectra calculation. Harmonic interatomic force constant is used as input *via* Phonopy code that is attained by density functional perturbation theory (DFPT).^45,46^ A 5 × 5 × 1 supercell with energy cut-off (500 eV) was used for phonon spectrum.

## Results and discussion

**Fig. 1:**
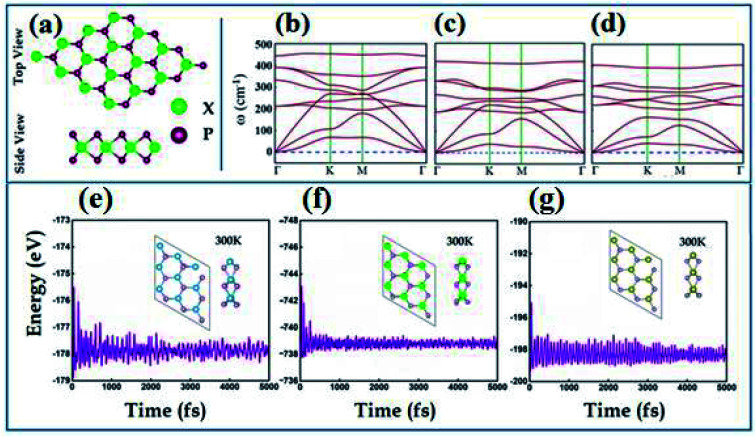
(a) Top and side view of XP_2_ monolayers, (b–d) phonon band structure and (e–g) AIMD calculated energy *versus* time plots with insets show the side and top view at 300 K of TiP_2_, ZrP_2_ and HfP_2_, respectively.

The transition metal dipnictides XP_2_ (X = Ti, Zr, and Hf) like transition metal dichalcogenides^47,48^ exhibit the same hexagonal structure having trigonal prismatic 2H phase with transition metal (X-atom) sandwiched between two phosphorus atoms as shown in Fig. 1(a). The optimized lattice parameters, bond lengths (*d*_X–P_), bond angle (*θ*_P–X–P_) and calculated band gap using PBE and HSE06 schemes are listed in Table 1. The phonon band spectrum of XP_2_ (X = Ti, Zr, Hf), displayed in Fig. 1(b–d), consists of three lowest frequency acoustic modes with no imaginary frequency at the Γ-point. This confirms that all studied monolayers are dynamically stable. Further, the thermal stability of all XP_2_ monolayers has been ascertained by performing *ab initio* molecular dynamics (AIMD) calculations at room temperature. The snap shots of the final geometry of XP_2_ monolayers with energy fluctuation *versus* time (5000 ps) have been shown in Fig. 1(e–g). All single layer XP_2_ have no considerable energy fluctuations and possess no broken bonds in the final structures at 300 K, indicating the experimental fabrication of understudy systems.

**Table 1:** The calculated lattice constant (*a* in Å), bond length (P–X in Å), bond angle (*θ*_P–X–P_ in degree) between X and P atoms, and band gap (*E*_g_ in eV) using PBE- and HSE06 functionals, of XP_2_ monolayers

Monolayer	*a* (Å)	P–X (Å)	(*θ*_P–X–P_)	*E*_g-PBE_ (eV)	*E*_g-HSE06_ (eV)
TiP_2_	3.80	2.47	54.73°	0.70	1.72
ZrP_2_	4.10	2.63	51.99°	0.67	1.43
HfP_2_	4.00	2.59	53.93°	1.23	2.02

**Fig. 2:**
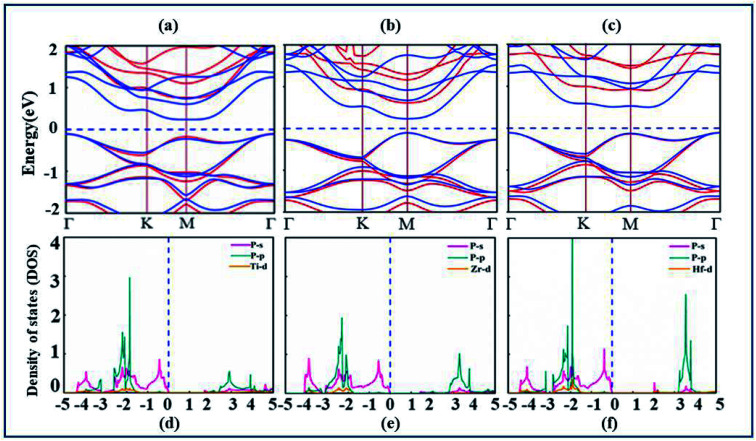
(a–c) The calculated electronic band structure represented by blue solid lines (PBE-functional) and red solid lines (HSE06 functional), and (d–f) partial density of states (PDOS) of TiP_2_, ZrP_2_ and HfP_2_, respectively.

After confirmation of structural stability and manufacturing possibility of monolayers XP_2_, electronic band structure and density of states (DOS) are calculated (as displayed in Fig. 2(a–c)) to gain deep insight into their electronic properties. According to our calculations, XP_2_(X = Ti, Zr, Hf) monolayers reveal semiconducting behavior with indirect band gap of 0.70 eV, 0.67 eV and 1.23 eV respectively with PBE functional. In addition, the hybrid functional (HSE06)^44^ is used to make correction to the self-interaction (SIF) error which underestimates band gap. The calculated band structure of XP_2_ monolayers exhibits the same band character with larger band gap values 1.72 eV (TiP_2_), 1.43 eV (ZrP_2_) and 2.02 eV (HfP_2_). Red and blue colors represent HSE06 and PBE band structure as shown in Fig. 2(a–c). For TiP_2_ and ZrP_2_systems, the conduction band minimum (CBM) lies at the M-point and valence band maximum (VBM) lies at the Γ-point of the Brillouin zone, representing an indirect band nature. In case of HfP_2_, CBM lies between M and Γ point while VBM lies at Γ-point of the Brillouin zone indicating an indirect semiconducting behavior. Similar trend is observed for other two dimensional materials.^25,28,49,50,52,54^

The analysis of individual states contributing near the Fermi level (*E*_F_) is obtained by plotting the partial density of states (PDOS) of understudy systems. The X-d (orange), P-s (pink), P-p (cyan) orbitals and Fermi level (*E*_F_) represented by blue dashed line are shown in Fig. 2(d–f). The PDOS of X-s and X-p states found deeper in energy region are avoided here. It is obvious from Fig. 2(d–f) that both VBM and CBM are predominantly attributed by P-s orbital of all XP_2_ systems.

**Fig. 3:**
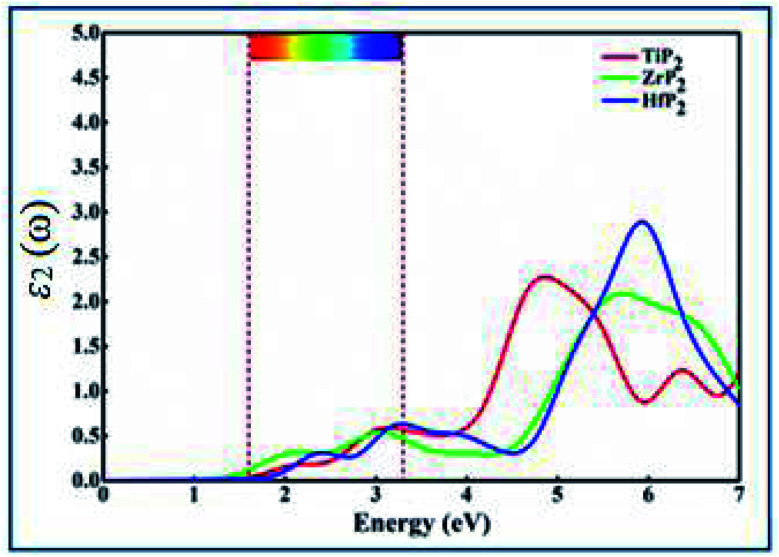
Imaginary part of the dielectric constant (*ε*_2_(*ω*)) as a function of photon energy for the XP_2_ (X = Ti, Zr, and Hf) monolayer obtained from the average of *x*, *y*, and *z* polarization vectors. The area between the two brown dashed lines is the energy range of visible light.

The optical behavior of XP_2_ systems is crucial for exploring the absorption and conversion of sunlight into electric current for various applications. Complex dielectric function specifically describes optical properties by formula *ε*(*ω*) = *ε*_1_(*ω*) + *iε*_2_(*ω*).^49^ In the present work, the optical absorption spectra in terms of imaginary part *ε*_2_(*ω*) is calculated between 0–7.0 eV energy range, as shown in Fig. 3. From *ε*_2_(*ω*) spectra of all XP_2_ systems, a considerable visible light absorption is found between 1.65–3.3 eV energy range, suggesting these monolayers as promising candidates for solar energy absorber and photovoltaic applications.

**Fig. 4:**
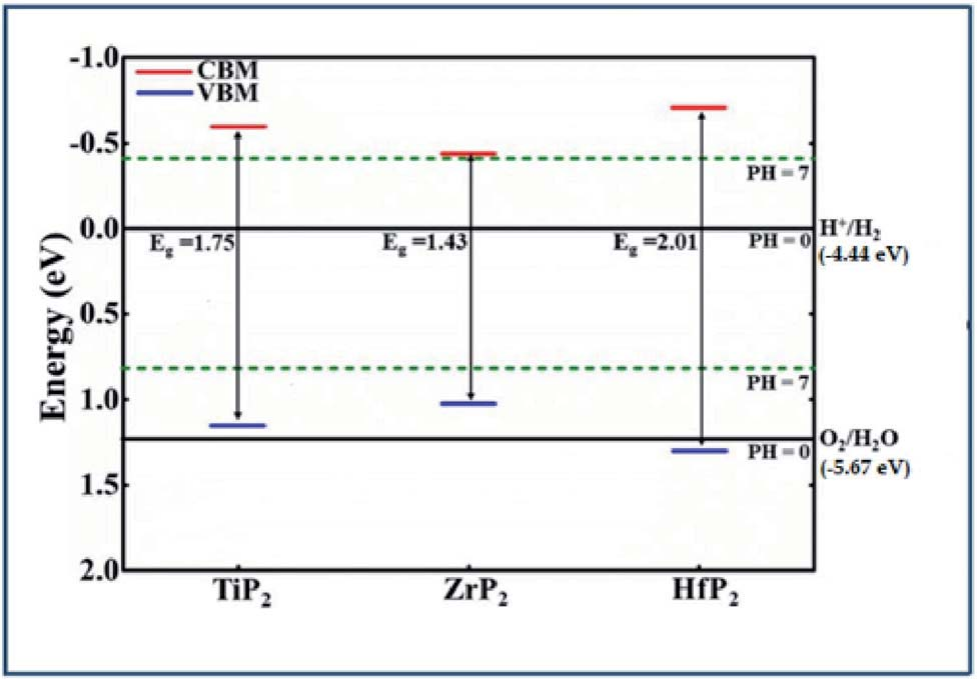
Band edge locations of the three of XP_2_(X = Ti, Zr, and Hf) monolayers relative to the water oxidation (O_2_/H_2_O) and reduction (H^+^/H_2_) potentials at pH = 0 and 7 levels.

Generally, the band gap of a material must be larger than 1.23 eV to perform the photocatalytic activity for water splitting. Using HSE06 functional, the calculated band gap values of XP_2_ monolayers exceed free energy value (1.23 eV), implying that XP_2_ monolayers satisfies the water splitting reaction. The valence band edge (*E*_VB_) and conduction band edge (*E*_CB_) positions are obtained by using HSE06 functional, schematically represented in Fig. 4. The standard redox potentials for photocatalytic water splitting mechanism are calculated by:

*E*_O2/H2O_ = −5.67 eV + pH × 0.059 eV and *E*_H^+^/H2_ = −4.44 eV + pH × 0.059 eV.^49–53^ Therefore, we considered redox potential levels for water as shown in Fig. 4. For HfP_2_ monolayer, the band edge potentials (both *E*_VB_ and *E*_CB_) straddle the standard redox potentials at pH = 0. However, the valence band edge of TiP_2_ and ZrP_2_ monolayers found above the standard oxidation potential thus, fails to perform oxidation evolution reaction at pH = 0. More interestingly, all XP_2_ monolayers with both *E*_VB_ and *E*_CB_ located below and above the standard redox potentials at pH = 7, indicates the ability to dissociate water into H^+^/H_2_ and O_2_/H_2_O under the irradiation of sun light. Similar behavior has also been reported in other literature.^27,49–54^ These findings predict XP_2_ monolayers as efficient candidates for photocatalysis and photovoltaic applications.

## Conclusion

In summary, the first principles calculations are performed to investigate the structural, electronic, optical properties and photocatalytic activity of transition metal dipnictides XP_2_ (X = Ti, Zr, Hf) monolayers. All single layer systems are dynamically and thermally stable. Two-dimensional TiP_2_, ZrP_2_ and HfP_2_ are indirect semiconductors (with band gap values 1.72 eV, 1.43 eV and 2.02 eV, respectively) with both VBM and CBM mainly attributed by P-s state. A considerable absorption of solar light is found between 1.65–3.3 eV. More interestingly, all XP_2_ systems (at pH = 7) and HfP_2_ monolayer (at pH = 0) are capable to perform redox reactions of water splitting. These results pave the path for designing future optoelectronic and photovoltaic devices.

## Conflicts of interest

There is no conflict of interest.

## Supplementary Material
